# Current News

**Published:** 1891-05

**Authors:** 


### Current News.

### THE DENTAL PROTECTIVE ASSOCIATION OF THE UNITED STATES.

*The impending suit of the Tooth Crown Company in the Supreme Court of the United States will be reached this week; therefore this communication should be read at once by every dentist in the country.*

THE purpose and plans of the Dental Protective Association of the United States have already been explained to you, and are, in brief, to defend its members, without expense to them when we have control of the case, in any suit brought against them under unjust claims of letters patent, owned by any corporation, party, or person.

The object of this circular is to show briefly why any dentist, not a member of this Association, must become so *on or before the 15th day of May next*,—*i.e.*, May 15, 1891,—or be cut off from any opportunity of joining the Association, or from any protection by the Association, in case he is sued under certain letters patent, owned by the International Tooth Crown Company, covering dental work now commonly known as "Richmond Crown." Such letters patent are now in suit in the Supreme Court of the United States, under a record made, and suit tried by a committee of dentists headed by Dr. A. L. Northrop, of New York City. This case will be tried at Washington, D. C., in a few days.

The Dental Protective Association of the United States had nothing to do with the making up of this record, but has put in its patent counsels, Messrs. Offield, Towle, and Linthicum, of Chicago, Ill., to argue the case in behalf of the dentists, with the attorney for Dr. A. L. Northrop's committee, Mr. J. K. Beach, of New Haven, Conn.

The International Tooth Crown Company and its counsel speak confidently of winning this case, and if they do they will undoubtedly sue every dentist in the United States who is not a member of our Association.

We do not believe that any member of our Association will be sued by the Crown Company, even if it win this case. It has not for the last two years sued any member of the Association on any of the forty or fifty patents owned by it, out of the State of New York. This company has been forced by the Association to dismiss every case it has ever brought against dentists in all parts of the United States, except the State of New York.

Our Association has sufficient proof and evidence in its possession to defeat the Richmond Crown patent in any suit brought against it, even if these patents are now sustained in the Supreme Court of the United States on the present record, but it is not justice to any of its present members that the Association receive or defend any dentist who remains outside until he is sued.

As has been already stated, the benefit of organization is that we can now step forward and defend any member, however humble or obscure, or however remote from the large centres, in any court, on short notice, with full and complete testimony to defeat these Tooth Crown patents, irrespective of and additional to any evidence presented in the case now pending in the United States Supreme Court.

The defence of one of these cases, which no one individual could afford, costs the Association many thousands of dollars and great additional expenditure of time, thought, and energy on the part of your committee; therefore, you can readily see the injustice of a dentist withholding or refusing to pay ten dollars *until he is sued*, and then expecting the Association to defend him.

We do not put our patent counsel in the argument of the case in the Supreme Court of the United States with the expectation of entire success upon the record now given, as it is in many respects unsatisfactory to us, but we do so in order to avert other dangers which we cannot explain here, and if possible to save the entire case. As before said in former circulars, these records cannot now be altered, but the accumulated evidence in our possession will enable us to bring much stronger testimony in a new suit.

1. Remember that the profession has already paid unjustly millions of dollars to the old Rubber Company.

2. Remember that the International Tooth Crown Company owns forty or fifty patents.

3. Remember that this is not the only patent company from which the Dental Protective Association has saved you.

4. Remember that the Crown Company will probably win the Crown suit as appealed. That a decision from the Supreme Court in favor of the Crown Company does not prevent the Protective Association from trying the same case over again on new evidence.

5. Remember we give you until May 15 to decide which side you will take, that of the dentists of America, or that of the Tooth Crown Company and other patent claimants.

6. Remember the membership fee is only ten dollars and no annual dues.

7. Remember to SIGN THE BY-LAWS AND SEND WITH YOUR CHECK *or note,* and thus save extra stamp and additional correspondence.

8. Remember we would rather have the money than the note, but when this is impossible, send the note payable when due.

9. Remember the funds accumulated are in charge of our treasurer, Lyman J. Gage, President of the First National Bank of Chicago.

10. Remember there are no salaries paid to officers.

11. Remember that united effort means success in its largest sense.

ARE YOU GOING TO HELP US?

All communications should be sent to the chairman, J. N. Crouse, 2231 Prairie Avenue, Chicago, Ill.

(This communication is sent to members as well as non-members. Members should use their utmost endeavors to secure new members.)

---

PENNSYLVANIA AND NEW JERSEY STATE DENTAL SOCIETIES JOINT UNION MEETING.—The Twenty-third Annual Meeting of the Pennsylvania State Dental Society and the Twenty-first Annual Meeting of the New Jersey State Dental Society will be held in joint session at Asbury Park, the famous New Jersey watering-place, on Thursday and Friday, July 16 and 17. The Papers will be by the most eminent men in the profession, who are already engaged. A great feature will be made of Clinics and Exhibits. Due notice of hotel and railroad rates, time-tables, etc., will be given in June.

CHARLES A. MEEKER, D.D.S.,
*Secretary New Jersey State Dental Society.*
H. NEWTON YOUNG, D.D.S.,
*Corresponding Secretary, Pennsylvania State Dental Society.*

---

UNIVERSITY OF MARYLAND, DEPARTMENT OF DENTAL SURGERY.—The Annual Commencement took place Wednesday, March 18, 1891. Number of graduates, 64; number of matriculates, 163.

